# Causal effects of immune cells in glioblastoma: a Bayesian Mendelian Randomization study

**DOI:** 10.3389/fneur.2024.1375723

**Published:** 2024-04-29

**Authors:** Mingsheng Huang, Yiheng Liu, Jie Peng, Yuan Cheng

**Affiliations:** ^1^Department of Neurosurgery, Second Affiliated Hospital, Chongqing Medical University, Chongqing, China; ^2^Department of Cardiology, Second Affiliated Hospital, Chongqing Medical University, Chongqing, China

**Keywords:** glioblastoma, immunity, causal inference, MR analysis, Bayesian analysis

## Abstract

**Background:**

Glioblastoma (GBM) is a highly malignant brain tumor, and immune cells play a crucial role in its initiation and progression. The immune system's cellular components, including various types of lymphocytes, macrophages, and dendritic cells, among others, engage in intricate interactions with GBM. However, the precise nature of these interactions remains to be conclusively determined.

**Method:**

In this study, a comprehensive two-sample Mendelian Randomization (MR) analysis was conducted to elucidate the causal relationship between immune cell features and the incidence of GBM. Utilizing publicly available genetic data, we investigated the causal associations between 731 immune cell signatures and the risk of GBM. Subsequently, we conducted a reverse Mendelian randomization analysis to rule out reverse causation. Finally, it was concluded that there is a unidirectional causal relationship between three subtypes of immune cells and GBM. Comprehensive sensitivity analyses were employed to validate the results robustness, heterogeneity, and presence of horizontal pleiotropy. To enhance the accuracy of our results, we concurrently subjected them to Bayesian analysis.

**Results:**

After conducting MR analyses, we identified 10 immune phenotypes that counteract glioblastoma, with the most protective being FSC-A on Natural Killer T cells (OR = 0.688, CI = 0.515–0.918, *P* = 0.011). Additionally, we found 11 immune cell subtypes that promote GBM incidence, including CD62L– HLA DR++ monocyte % monocyte (OR = 1.522, CI = 1.004–2.307, *P* = 0.048), CD4+CD8+ T cell % leukocyte (OR = 1.387, CI = 1.031–1.866, *P* = 0.031). Following the implementation of reverse MR analysis, where glioblastoma served as the exposure variable and the outcomes included 21 target immune cell subtypes, we discerned that only three cell subtypes (CD45 on CD33+ HLA DR+ CD14dim, CD33+ HLA DR+ Absolute Count, and IgD+ CD24+ B cell Absolute Count) exhibited a unidirectional causal association with glioblastoma.

**Conclusion:**

Our study has genetically demonstrated the close relationship between immune cells and GBM, guiding future clinical research.

## Introduction

Glioma is the most prevalent form of primary malignant tumor of the central nervous system with an incidence of 5.6/100,000 per year in adults (1). The most aggressive subtype of glioma is glioblastoma (GBM), currently classified as grade 4 astrocytoma with a mutation in the isocitrate dehydrogenase gene (IDH) according to The World Health Organization (WHO) (2). Surgical resection of the tumor followed by radiotherapy and chemotherapy with temozolomide is a common GBM treatment method. Despite comprehensive treatment advances, GBM remains one of the deadliest human cancers due to its high recurrence rate and therapy resistance. Highly invasive nature, high heterogeneity, and immune evasion are regarded as pivotal determinants linked to treatment failure and disease relapse in GBM (3, 4). Overall, the prognosis of GBM is extremely poor, with a 5-year survival rate of <5%. It causes a heavy burden on families and society (5). Recently, immunotherapy has provided a new method to cure this disease. This primarily encompasses immune checkpoint inhibitors, personalized vaccines, Chimeric Antigen Receptor T (CAR-T) cell therapy, immune cell therapy, and other methodologies (6, 7). However, GBM is a highly immunosuppressive tumor with several immune escape mechanisms present (8, 9). Although immunotherapy has provided a new approach for treating glioblastoma, the lack of large-scale clinical randomized controlled trials to validate its efficacy and safety is attributed to ethical considerations and other factors. Moreover, the intricate relationship among immune cells, immunosuppressive cells, inflammatory responses, and the occurrence, development, and recurrence of glioblastoma is highly complex, making it challenging to arrive at a definitive conclusion regarding their interplay (10, 11). Microglia, as the indigenous macrophages of the central nervous system, are collectively known as tumor-associated macrophages (TAMs), forming the primary barrier of innate immunity within the central nervous system (12). TAMs adjust their phenotypes in response to the stimuli encountered within their microenvironment. Traditionally, two TAM phenotypes have been delineated: M1 macrophages, characterized by pro-inflammatory and anti-tumor properties, and M2 macrophages, which exhibit anti-inflammatory and pro-tumor characteristics (13). TAMs are a major type of immune cells in the tumor microenvironment. However, there is controversy surrounding the role of TAMs in glioblastoma. Some studies suggest that TAMs may promote the growth, invasion, and metastasis of glioblastoma, while others indicate that TAMs may counteract tumor growth (14–16). Furthermore, T cells constitute the principal lymphocytic constituent of the glioblastoma tumor microenvironment (TME), exerting both pro-tumor and anti-tumor functions. Various subsets of T cells can be discerned, including Cluster of Differentiation 4+ T (CD4+ T) helper cells, CD8+ cytotoxic T cells, and regulatory T cells (17). However, the role of T cells is also controversial. Some studies suggest that T cells can recognize and attack tumor cells, thereby combating tumor growth, while others have found that T cells may be suppressed by the tumor cells' immune evasion mechanisms in gliomas (18–20). Natural killer cells, originating from the bone marrow, possess effector functions mediated by cytokine production and cytotoxic activity. Their efficacy is often modulated by immunosuppressive factors released by tumor cells (21). Traditionally, microglial cells have been regarded as the immune cells of the central nervous system and may potentially counteract tumor growth. However, recent studies have suggested that in certain circumstances, microglial cells may promote the growth and metastasis of gliomas rather than inhibit them. This finding has sparked further debate regarding the functional role of microglial cells in gliomas (22, 23). While the roles of some immune cells in GBM have been elucidated, the diverse subtypes of immune cells contribute to ongoing research and controversies in the field. Therefore, further research is required to elucidate the roles of different subtypes of immune cells in glioblastoma.

Mendelian randomization (MR), a causal inference method, has been extensively applied in genetic epidemiology (24). In contrast to traditional observational studies, MR, utilizing genetic variation as instrumental variables (IVs), stands as a widely acknowledged approach to alleviate potential confounding factors (25). This method effectively navigates around issues related to reverse causation and confounding factors, enabling a more precise inference of the causal relationship between exposure and outcome. The rationality of the causal sequence in MR is of utmost importance. Previous observational studies have identified numerous associations between immune cell features and glioblastoma, validating the hypothesis of their correlation (26–28). In this study, a comprehensive two-sample MR analysis was conducted to ascertain the causal relationship between different immune cell subtypes and GBM.

## Materials and methods

### Study design

**Figure 1:**
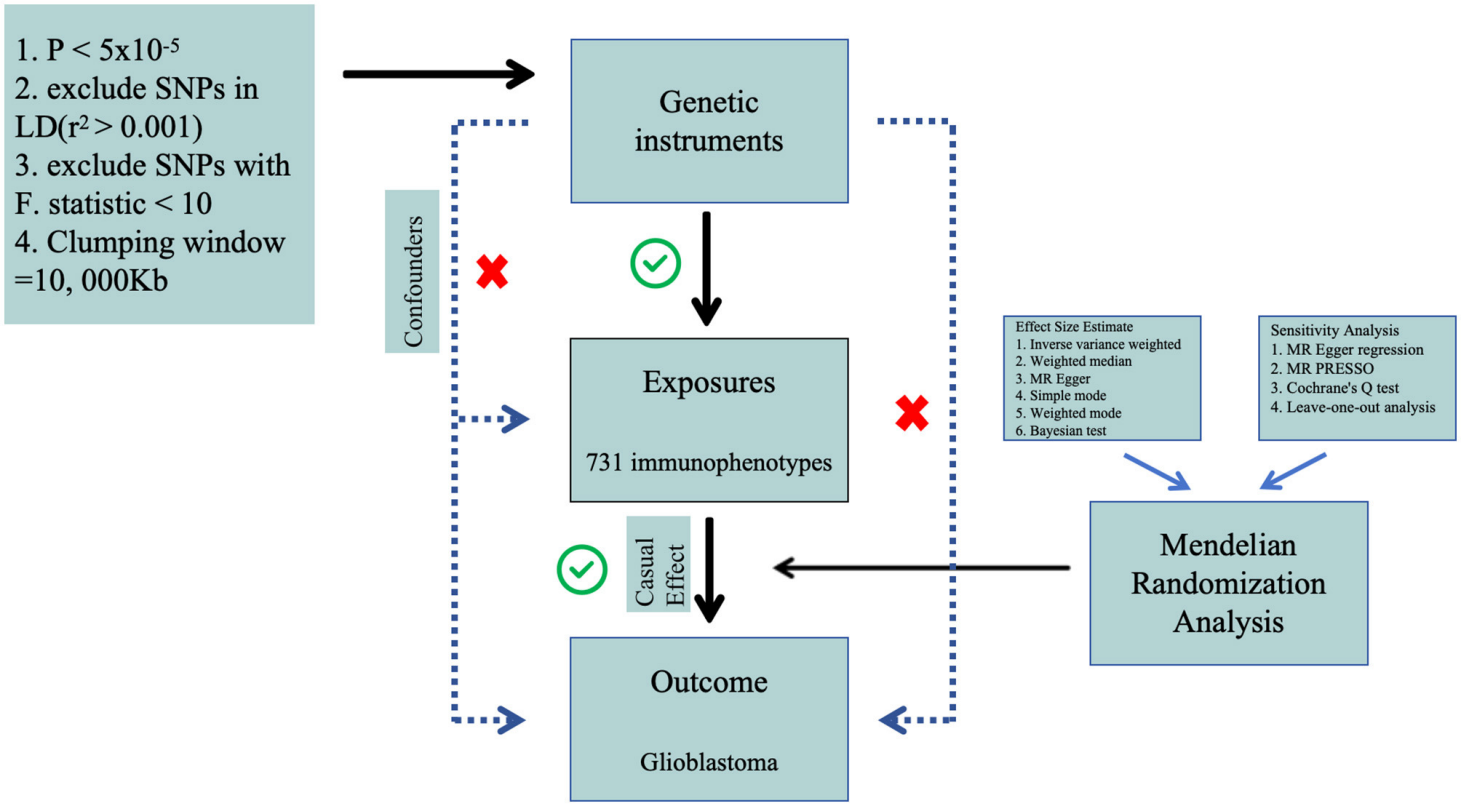
The flow diagram of Mendelian randomization analysis.

We performed a two-sample Mendelian Randomization (MR) analysis to evaluate the causal relationship between 731 immune cell features (categorized into seven groups) and glioblastoma. MR employs genetic variations as proxies for risk factors, and thus, valid instrumental variables in causal inference must meet three essential assumptions: (1) genetic variations are directly associated with the exposure; (2) genetic variations are not correlated with potential confounders between the exposure and outcome, and (3) genetic variations do not influence the outcome through pathways other than the exposure (Figure 1). The research investigations included in our analysis received approval from the respective institutional review boards, and participants provided informed consent.

### Data sources

The GWAS summary statistics for glioblastoma (finngen_R10_C3_GBM_EXALLC) were sourced from FinnGen Database R10, including 253 cases of brain glioblastoma and 314,193 controls. The FinnGen project has amassed biological specimens and clinical data from more than 300,000 individuals in Finland. This dataset encompasses diverse data types, including genomic data, clinical diagnoses, biological sample sequencing, and medical records. Through the analysis of extensive genetic and clinical data, the project seeks to unveil associations between genes and various diseases, alongside the impact of environmental and lifestyle factors on these relationships. Its overarching goal is to elucidate the interplay between genes and health. As a publicly accessible resource, it can be accessed via the website (https://www.finngen.fi/en) (29).

GWAS summary statistics for each immune trait are publicly available from the GWAS Catalog (accession numbers within the range of GCST0001391 to GCST0002121) (30). The data contain 731 immunophenotypes, with categories such as absolute cell (AC) counts (*n* = 118), median fluorescence intensities (MFI) reflecting surface antigen levels (*n* = 389), morphological parameters [MP] (*n* = 32), and relative cell (RC) counts (*n* = 192). These features encapsulate various immune cell types, including B cells, conventional dendritic cells (CDCs), mature stages of T cells, monocytes, myeloid cells, TBNK (T cells, B cells, natural killer cells), and Treg panels. The initial GWAS analyses involved a cohort of 3,757 individuals of European descent, thereby ensuring a comprehensive and diverse representation of the datasets. Utilizing high-density arrays, genotyping was performed on an extensive set of around 22 million single nucleotide polymorphisms (SNPs). Subsequently, imputation was carried out employing the Sardinian sequence-based reference panel. Covariate adjustments, specifically accounting for sex, age, and age squared, were systematically incorporated into the association analyses (31). This rigorous methodology aimed to enhance the accuracy and reliability of the research findings while minimizing the risk of confounding factors.

### Selection of instrumental variables (IVs)

In both forward MR studies (with immune cells as exposure and GBM as outcome) and reverse MR studies (with GBM as exposure and immune cells as outcome), we employed identical methodologies for experimentation. In the initial phase, single nucleotide polymorphisms associated with exposure were judiciously selected based on a genome-wide significance threshold (*P* < 5 × 10^−5^) in accordance with previous researches (30, 32). Subsequently, the independence of the chosen SNPs was assessed through pairwise linkage disequilibrium analysis, employing exclusion criteria for SNPs in linkage disequilibrium (*r*^2^ > 0.001 and a clumping window <10,000 kb) (33). Thirdly, the *F*-statistic was computed to ascertain the robustness of each SNP, with the exclusion of SNPs possessing an *F*-statistic <10 (34). A rigorous data harmonization process was implemented to ensure concordance between SNP effects on exposure and outcome, aligning with the same allele. The F-statistic for each SNP was calculated using the formula *F* = *R*^2^/(1–*R*^2^) × (*N* – 2), where *R*^2^ represents the variance of exposure explained by the instrumental variables (IVs), and N indicates sample size. The variance of exposure explained by the instrument variable was calculated with the formula *R*^2^ = β^2^/(β^2^ + se^2^ × *N*), in which β denotes the effect size for the genetic variant of interest, se represents the standard error for β, and *N* represents the sample size.

### Effect size estimate and sensitivity analysis

We employed the random-effect inverse variance-weighted (IVW) method as the primary analysis due to its robustness, providing a conservative estimate even in the presence of heterogeneity (35). Additionally, supplementary analyses were conducted employing the weighted median (WM) and MR-Egger methods to validate the robustness of the IVW estimates. MR-Egger regression served as a test for unbalanced pleiotropy and substantial heterogeneity (36). In the presence of pleiotropy, MR-Egger estimates were considered more persuasive than IVW estimates. Furthermore, when at least half of the weighted variance resulting from horizontal pleiotropy was valid, the WM estimates could provide robust effect estimates. In summary, a significant estimate consistently observed in the direction between IVW, WM, and MR-Egger was considered statistically significant.

We conducted a comprehensive set of sensitivity analyses, encompassing Cochran's *Q* tests, funnel plots, leave-one-out analyses, and MR-Egger intercept tests. Specifically, heterogeneity was assessed through Cochran's *Q* tests, and the intercept term derived from MR-Egger regression was employed to evaluate pleiotropy. Leave-one-out analyses were performed to determine whether the causal estimate was influenced by any single SNP. All analyses were executed using the “Two Sample MR” package (version 0.5.8) in R software (version 4.3.1). Statistical significance was defined at a two-sided *P*-value < 0.05. Effect estimates were reported as odds ratios (OR) per standard deviation (SD) increment of the corresponding exposure. To enhance the precision of our findings, we employed the coloc R package (https://chr1swallace.github.io/coloc/, version 5.1.0) for a Bayesian co-localization test on the MR results, enabling the estimation of the posterior probability associated with shared genetic variants (37).

## Results

We conducted a comprehensive MR investigation to explore the causal impact of genetically predicted 731 immunophenotypes on the morbidity of glioblastoma. In summary, we selected SNPs to genetically predict the causal influence of 731 immune cell types on GBM. The number of SNPs utilized in each MR analysis varied between 11 and 32. Notably, the *F*-statistic values for each genetic instrument surpassed 10, indicative of their robust instrumental strength.

### The causal effect between the immunophenotypes and glioblastoma

After conducting preliminary analyses on the associations between genetically instrumental immune cell features and the risk of glioblastoma mainly by IVW method, we identified causal associations for four groups of immune cells, comprising 21 distinct immune cell types including five were in the B cell panel, four in the CDC panel, two in the Maturation stages of T cell panel, three in the Myeloid cell panel, two in the Treg panel and five in the TBNK panel. We observed protective effects for 10 immunophenotypes against glioblastoma, while 11 immunological cell subtypes were found to promote its incidence. The most significant protective cell types are FSC-A on Natural Killer T cells (OR = 0.688, CI = 0.515–0.918, *P* = 0.011), CD38 on Plasma Blast-Plasma Cells (OR = 0.175, CI = 0.532–0.959, *P* = 0.025), CD3 on CD39+ resting CD4 regulatory T cells (OR = 0.720, CI = 0.584–0.889, *P* = 0.002), and CD20 on CD20-CD38- B cells (OR = 0.721, CI = 0.542–0.960, *P* = 0.025), respectively. While, the primary immune cell subtypes promoting the incidence of glioblastoma include CD62L– HLA DR++ monocyte % monocyte (OR = 1.522, CI = 1.004–2.307, *P* = 0.048), CD4+CD8+ T cell % leukocyte (OR = 1.387, CI = 1.031–1.866, *P* = 0.031), Lymphocyte Absolute Count (OR = 1.369, CI = 1.050–1.786, *P* = 0.020), Granulocyte Absolute Count (OR = 1.363, CI = 1.044–1.780, *P* = 0.023). The main results are presented in Table 1 and the detailed results are found in Supplementary Table S1.

**Table 1:** Causal effects of immune cells on GBM by IVW.

**Traits**	**Beta**	**OR**	**Low**	**Up**	***P*-value**
IgD+ CD24+ B cell absolute count	0.305	1.357	1.000	1.840	0.049
CD19 on IgD+ CD38dim B cell	0.127	1.136	1.015	1.270	0.025
CD19 on IgD+ CD24– B cell	0.116	1.122	1.003	1.256	0.043
CD20 on CD20– CD38– B cell	−0.327	0.721	0.542	0.960	0.025
CD38 on plasma blast-plasma cell	−0.336	0.715	0.532	0.959	0.025
CD62L– HLA DR++ monocyte %monocyte	0.420	1.522	1.004	2.307	0.048
CD86 on CD62L+ myeloid dendritic Cell	0.232	1.262	1.017	1.566	0.035
Myeloid dendritic cell absolute count	0.185	1.203	1.029	1.406	0.020
CD11c on monocyte	−0.203	0.816	0.667	1.000	0.049
CD3 on effector memory CD4+ T cell	−0.202	0.817	0.679	0.984	0.033
Effector memory CD4–CD8– T cell %CD4–CD8– T cell	−0.220	0.803	0.673	0.957	0.014
CD45 on CD33+ HLA DR+ CD14dim	0.268	1.307	1.072	1.595	0.008
CD33+ HLA DR+ absolute Count	−0.112	0.894	0.815	0.981	0.018
CD66b on CD66b++ myeloid cell	−0.213	0.808	0.685	0.952	0.011
CD4+CD8+ T cell %leukocyte	0.327	1.387	1.031	1.866	0.030
Lymphocyte absolute count	0.314	1.369	1.050	1.786	0.020
Granulocyte absolute count	0.310	1.363	1.044	1.780	0.023
CD8dim T cell %leukocyte	−0.230	0.795	0.633	0.997	0.047
FSC-A on natural killer T	−0.374	0.688	0.515	0.918	0.011
CD4 on activated & secreting CD4 regulatory T cell	0.141	1.152	1.007	1.317	0.039
CD3 on CD39+ resting CD4 regulatory T cell	−0.328	0.720	0.584	0.889	0.002

All P < 0.05.

### Bi-directional causal inference between glioblastoma and 21 target immune cell subtypes

**Figure 2:**
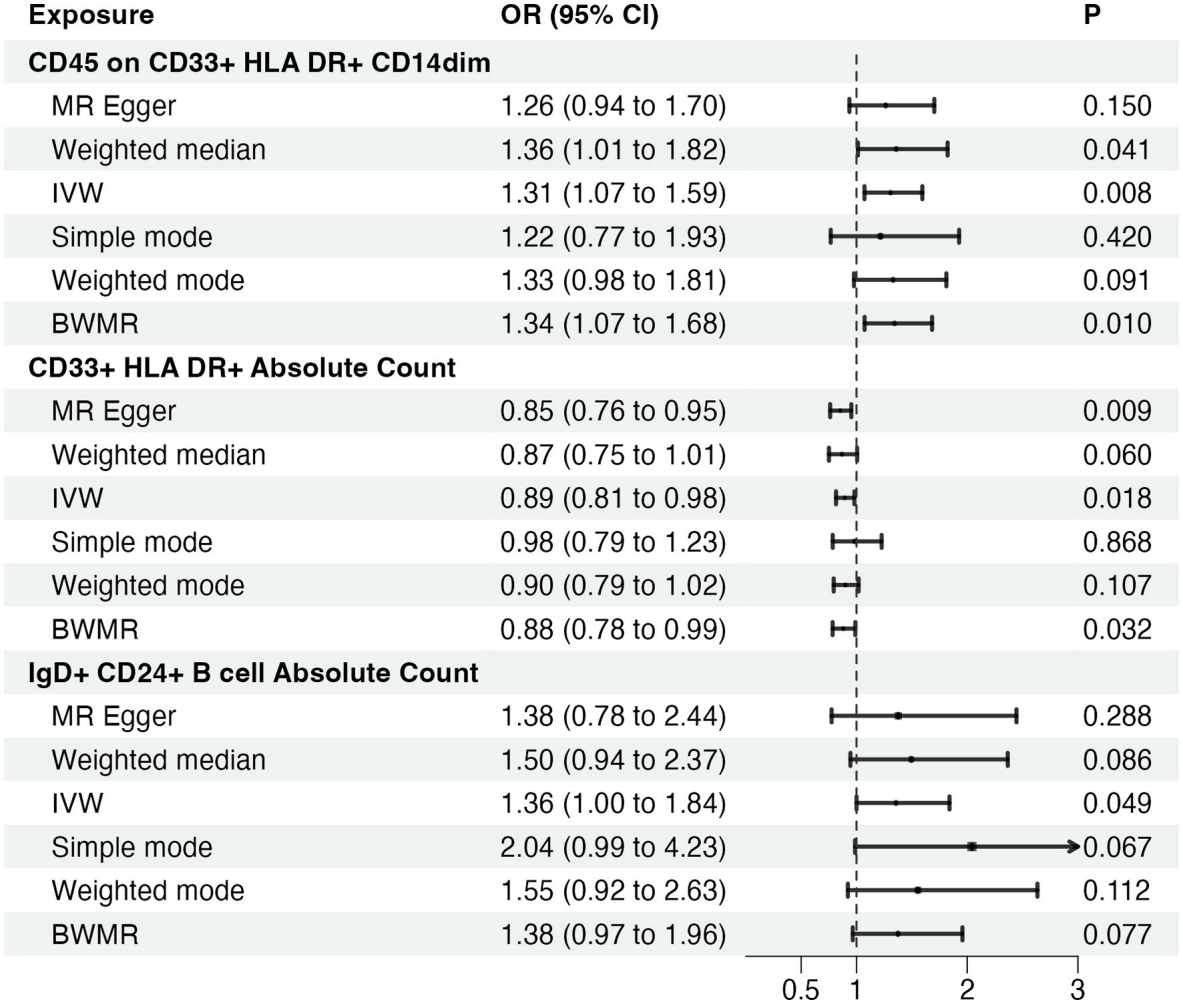
Causal effects of immune cells on glioblastoma. MR, Mendelian randomization; IVW, Inverse Variance Weighting; BWMR, Bayesian Mendelian Randomization.

**Figure 3:**
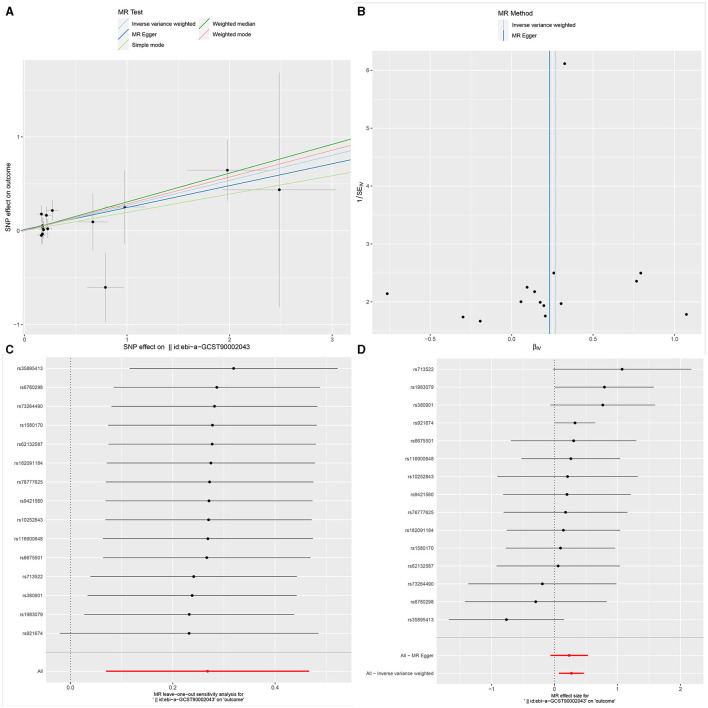
The Scatter plot, Funnel plot, leave-one-out sensitivity analysis, and Forest plot of CD45 on CD33+ HLA DR+ CD14dim.

**Figure 4:**
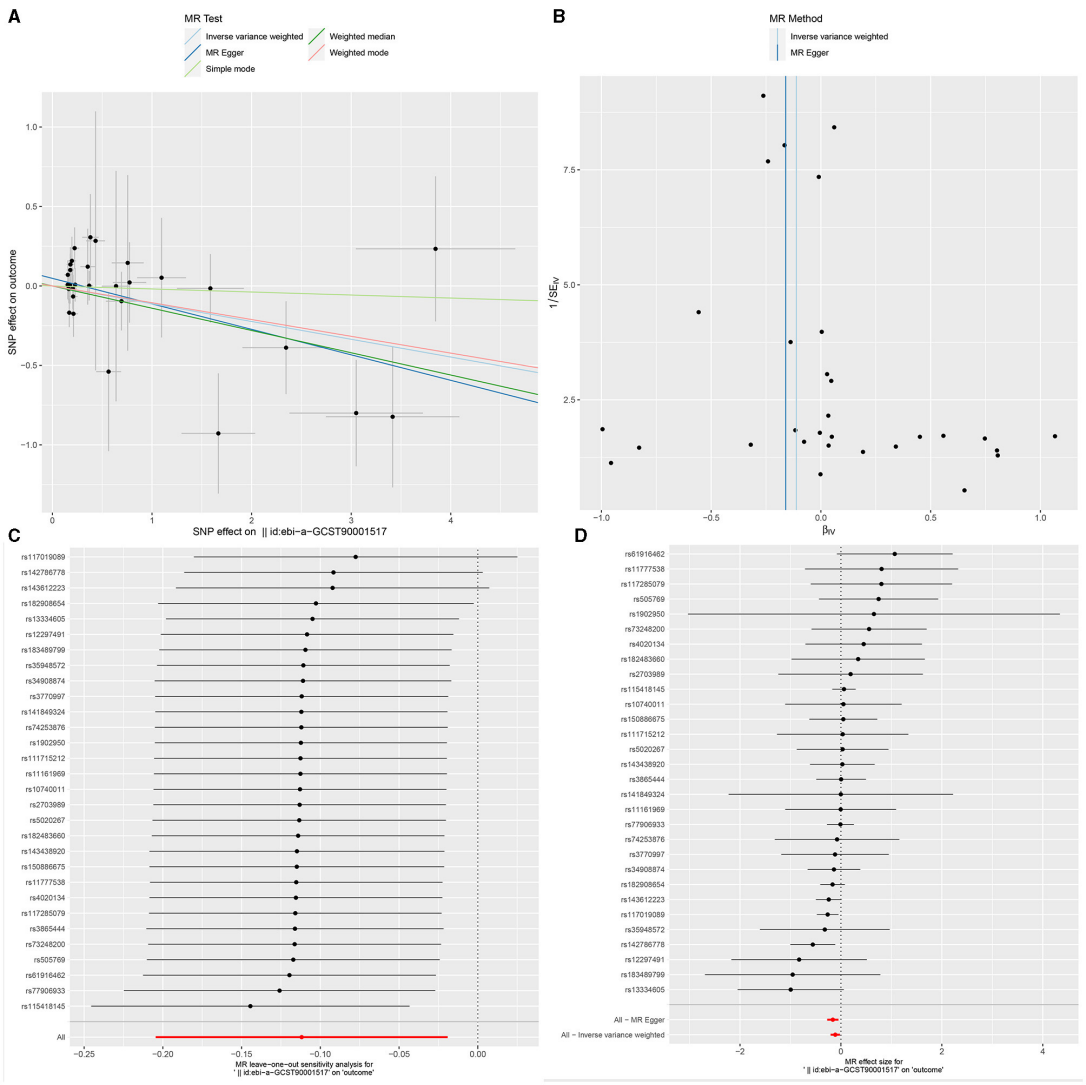
The Scatter plot, Funnel plot, leave-one-out sensitivity analysis, and Forest plot of CD33+ HLA DR+ Absolute Count.

**Figure 5:**
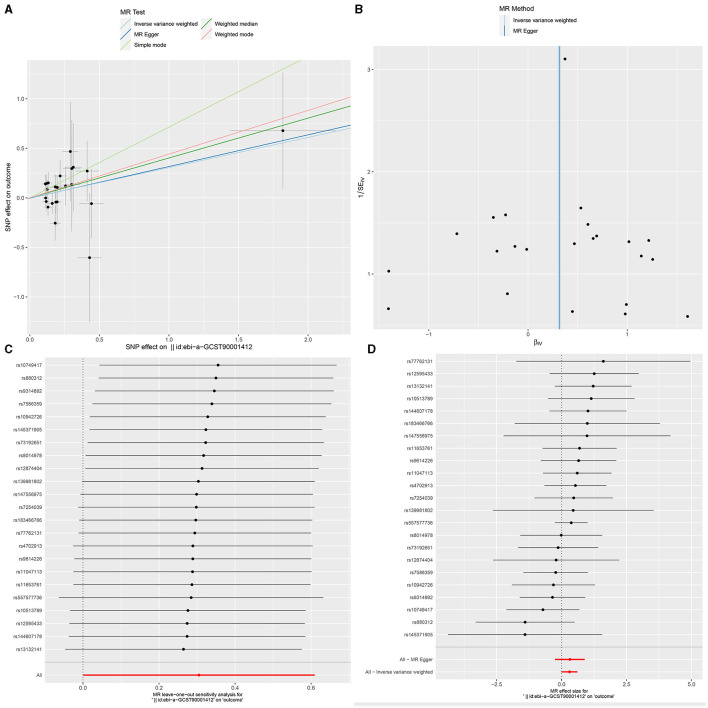
The Scatter plot, Funnel plot, leave-one-out sensitivity analysis, and Forest plot of IgD+ CD24+ B cell Absolute Count.

Given the observed statistically significant positive correlation, we deemed it essential to scrutinize the potential reverse association. The results of reverse Mendelian Randomization (MR) analysis indicate estimates of reverse causation effects. The reverse MR results reveal an inverse association between GBM and immune cells. The estimated effect of this reverse association is statistically significant (*P*-value < 0.05), suggesting a potential relationship between changes in GBM and variations in immune cells. To ensure accurate causal interpretation and enhance result reliability, our objective was to eliminate significant reverse associations during the analysis. Therefore, after conducting Mendelian Randomization analysis with glioblastoma as the exposure and the 21 target immune cell subtypes as outcomes, we identified only three cell subtypes with a unidirectional causal relationship with glioblastoma (CD45 on CD33+ HLA DR+ CD14dim, CD33+ HLA DR+ Absolute Count and IgD+ CD24+ B cell Absolute Count), and the OR measured by IVW method were OR = 1.307, CI = 1.072–1.595, *P* = 0.008 (Figures 3A, D), OR = 0.894, CI = 0.815–0.981, *P* = 0.018 (Figures 4A, D) and OR = 1.357, CI = 1.000–1.840, *P* = 0.049 (Figures 5A, D) respectively. The details of their effect estimates and confidence intervals, significance statements, and sensitivity analyses can be found in Table 2 and Figure 2. The complete dataset is available in the Supplementary Table S2.

**Table 2:** Mendelian Randomization assessments regarding the connection between genetically instrumented immune cells and GBM.

**Outcome**	**Exposure**	**Method**	**OR**	**95% CI**	***P*-value**
GBM	CD45 on CD33+ HLA DR+ CD14dim	MR Egger	1.263	(0.936–1.703)	0.150
		Weighted median	1.359	(1.013–1.823)	0.041
		IVW	1.307	(1.072–1.595)	0.008
		Simple mode	1.216	(0.767–1.927)	0.420
		Weighted mode	1.331	(0.977–1.812)	0.091
		BWMR	1.344	(1.073–1.683)	0.010
GBM	CD33+ HLA DR+ absolute count	MR Egger	0.852	(0.762–0.953)	0.009
		Weighted median	0.869	(0.751–1.006)	0.060
		IVW	0.894	(0.815–0.981)	0.018
		Simple mode	0.981	(0.785–1.227)	0.868
		Weighted mode	0.900	(0.794–1.019)	0.107
		BWMR	0.880	(0.784–0.989)	0.032
GBM	IgD+ CD24+ B cell absolute count	MR Egger	1.377	(0.775–2.445)	0.288
		Weighted median	1.495	(0.945–2.366)	0.086
		IVW	1.357	(1.000–1.840)	0.050
		Simple mode	2.043	(0.987–4.229)	0.067
		Weighted mode	1.555	(0.922–2.634)	0.112
		BWMR	1.375	(0.966–1.957)	0.077

### SNP selection

Finally, 16, 30, and 23 SNPs were identified as IVs for CD45 on CD33+ HLA DR+ CD14dim, CD33+ HLA DR+ Absolute Count, and IgD+ CD24+ B cell Absolute Count, respectively. Moreover, the *F*-statistics for all IVs > 10, indicate no evidence of weak instrumental bias. The details of these IVs are shown in Supplementary Tables S4–S6. Similarly, in the reverse MR analysis, 30 SNPs were identified as instrumental variables for GBM. Importantly, all calculated *F*-values exceeded 10, ranging from 16.47260513 to 27.68019885. This observation indicates that the selected SNPs effectively represent the exposure variable, thereby enhancing the reliability and interpretability of the results. Consequently, these results ensure the credibility of the causal inferences derived from the Mendelian randomization approach (Supplementary Tables S7–S9).

### Sensitivity analysis

Ultimately, sensitivity analyses were conducted for the results. No evidence of horizontal pleiotropy of exposure factors was detected when employing MR-Egger regression detection and the MR-PRESSO global test (*P* > 0.05; Figures 3B, 4B, 5B). Cochran's IVW *Q*-test results indicated no significant heterogeneity among IVs. For specific details refer to Table 3. Similarly, leave-one-out sensitivity analyses suggested that no individual SNP significantly influenced the causal association (Figures 3C, 4C, 5C).

**Table 3:** Assessment of diversity and directional pleiotropy employing various methodologies.

**Immune cell**	**Heterogeneity**	**Horizontal pleiotropy**	
	**Cochran's** ***Q P***	**MR-Egger intercept** ***P***	**MR-PRESSO global test** ***P***
CD45 on CD33+ HLA DR+ CD14dim	0.591	0.766	0.641
CD33+ HLA DR+ absolute count	0.550	0.140	0.550
IgD+ CD24+ B cell absolute count	0.828	0.953	0.844

## Discussion

Through a two-sample MR analysis, we explored the causal relationships between different immune cell subtypes and the onset of GBM. The results indicated inhibitory effects on its occurrence for 10 immune cell subtypes exemplified by FSC-A on Natural Killer T cells (OR = 0.688, CI = 0.515–0.918, *P* = 0.011). Conversely, ten subtypes, including Lymphocyte Absolute Count (OR = 1.369, CI = 1.050–1.786, *P* = 0.020), exhibited a promoting effect on GBM incidence. Subsequently, through reverse MR analysis, we identified three distinct subtypes exhibiting singular causal relationships with GBM.

Our study revealed a decreased risk of GBM with an elevated mean fluorescence intensity of CD45 on CD33+ HLA DR+ CD14dim (Maturation stages of T cell panel). CD45 on CD33+ HLA DR+ CD14dim, where HLA DR is a component of the major histocompatibility complex (MHC) class II molecules encoded by the human leukocyte antigen complex on chromosome 6 region 6P21. CD45, a phosphatase typically expressed on the surface of leukocytes, especially immune system cells, plays a crucial role in regulating cell signaling and immune cell activity. CD33, a cell surface molecule commonly expressed in myeloid cells, particularly in the early stages of myeloid cell development, is involved in cell adhesion and immune regulation. CD14 is a surface marker typically found on monocytes and macrophages. In summary, CD45 on CD33+ HLA DR+ CD14dim describes a myeloid cell subtype characterized by surface markers CD45, CD33, HLA DR, and CD14dim. Through dephosphorylation and phosphorylation processes, CD45 plays a crucial role in regulating cell signaling, participating in cell activation and signal transduction. Its significance is particularly pronounced in the modulation of T-cell receptor (TCR) signal transduction. It aids in ensuring that T cells can undergo appropriate activation responses when stimulated by antigens.

The incidence of GBM correlates positively with the augmentation of CD33+ HLA DR+ Absolute Count (Myeloid cell panel). Previous research has indicated that myeloid cells are commonly observed within the tumor microenvironment (TME), undergoing polarization that includes myeloid-derived suppressor cells (MDSCs), tumor-associated macrophages, and microglia (TAMs), tumor-associated neutrophils (TANs), and tumor-associated dendritic cells (TADCs). This polarization serves to enhance both tumorigenesis and immune suppression (38, 39). CD33 is a cell surface molecule, characterized as a glycoprotein, and is involved in the development and regulation of immune cells. CD33 may play a role in immune modulation, with some studies suggesting its involvement in immunosuppression, including the inhibition of excessive immune activation. In the context of GBM, this regulatory function could impact the activity of immune cells, thereby influencing the immune response against the tumor. Additionally, there is evidence indicating that CD33 may contribute to anti-tumor immune responses. In certain scenarios, inhibiting CD33 has been proposed as a strategy to enhance the immune system's response to tumors. However, due to the highly heterogeneous nature of glioblastoma, characterized by variations in immune features and treatment responses among individuals, further in-depth experimental and clinical research is required to ascertain the precise role of CD33 in GBM.

A similar trend was observed in IgD+ CD24+ B cell Absolute Count (B cell panel), suggesting that an increase in the absolute count of these cells was associated with a higher risk of GBM. CD24 is a cell surface molecule involved in cell adhesion, signal transduction, and immune regulation. Within B cells, the expression of CD24 is likely associated with cellular differentiation and function. In the interaction between the immune system and the tumor microenvironment, these cells may play a distinct role. In certain instances, specific B cell subpopulations may participate in tumor immune evasion by modulating immune responses or promoting immune tolerance. This could contribute to the tumor's ability to evade immune surveillance. Simultaneously, B cells may influence the immune characteristics of the tumor microenvironment through the secretion of cytokines, antibodies, or other molecules. This influence could impact the growth and development of the tumor. Furthermore, the interaction between B cells and T cells may play a crucial role in immune responses. In the field of tumor immunology, B cells may influence anti-tumor immune responses through their interactions with T cells (40, 41). Therefore, a comprehensive understanding of the role of IgD+ CD24+ B cells in GBM requires further experimental and clinical research.

Our study employed a MR design to investigate the causal effects of different immune cell subtypes on GBM. Because Mendelian Randomization utilizes natural genetic variation as a random allocation factor, based on the natural allocation of individual genetic variation, it reduces the influence of confounding factors and reverse causation. It has the advantage of simulating a randomized controlled trial, with lower costs and usually more ethically acceptable, as it does not require active intervention on participants and does not involve risks to individual health. However, it is important to acknowledge several limitations. Potential heterogeneity and horizontal pleiotropy were not comprehensively assessed, and the majority of GBM patients in our analysis were of European ancestry, thus limiting the generalizability of our findings and requiring validation across different populations. Furthermore, our GBM cases were sourced from public databases, with a relatively small sample size of only 253 cases, which may impact the robustness of our results. Additionally, we initially set the threshold for selecting single nucleotide polymorphisms associated with immune cells and GBM as exposures at *P* < 5 × 10^−8^. However, due to limited availability of such SNPs, we widened the threshold to *P* < 5 × 10^−5^, potentially introducing some instability into the results. Future research efforts could expand the sample range to encompass populations of various ethnic backgrounds and geographical regions to confirm the universality and reliability of the findings.

## Conclusion

In summary, our comprehensive bidirectional Mendelian Randomization (MR) analysis has demonstrated the causal associations between multiple immunophenotypes and glioblastoma (GBM), highlighting the intricate pattern of interactions between the immune system and GBM. Moreover, our study significantly mitigated the impact of unavoidable confounding factors, reverse causality, and other influences. This may provide a novel avenue for researchers to explore immunotherapeutic interventions for glioblastoma, prompting discussions on early interventions and treatment strategies.

## Perspectives

Based on the aforementioned discussions, our findings offer several avenues for future research. Firstly, it is imperative to incorporate a larger sample size to validate and replicate our results across diverse populations, ensuring their robustness and generalizability. Secondly, further investigations are warranted to elucidate the specific mechanisms and signaling pathways underlying the potential roles of different immune cell subtypes in glioblastoma pathogenesis. Thirdly, given the current focus on immunotherapy for glioblastoma, clinical trials assessing the therapeutic potential of immune modulation targeting newly identified immune cells may hold promise for improving patient outcomes. Lastly, embracing precision medicine approaches and integrating genetic, immunological, and clinical data into predictive models can optimize personalized treatment strategies for glioblastoma patients. These research directions are crucial for advancing our understanding of the intricate interplay between immune cell subtypes and glioblastoma, ultimately leading to enhanced diagnostic and therapeutic interventions for this devastating disease.

## Data availability statement

The original contributions presented in the study are included in the article/Supplementary material, further inquiries can be directed to the corresponding author.

## Ethics statement

Ethical review and approval was not required for the study on human participants in accordance with the local legislation and institutional requirements. Written informed consent from the patients/participants or patients/participants legal guardian/next of kin was not required to participate in this study in accordance with the national legislation and the institutional requirements.

## Author contributions

MH: Writing – original draft, Writing – review & editing. YL: Conceptualization, Data curation, Formal analysis, Writing – review & editing. JP: Funding acquisition, Methodology, Writing – review & editing. YC: Writing – review & editing, Conceptualization, Methodology.
